# Chondromesenchymal hamartomas in a 24-year-old male mimicking a posterior mediastinal tumor and a 5-month-old boy with postoperative disseminated intravascular coagulation: two case reports

**DOI:** 10.1186/s13000-020-00940-0

**Published:** 2020-05-12

**Authors:** Yue Li, Danyang Zheng, Min Zuo, Yang Li, Huizhong Zhang

**Affiliations:** 1 State Key Laboratory of Oncology in South China, Collaborative Innovation Center for Cancer Medicine, Sun Yat-sen University Cancer Center, Guangzhou, 510060 China; 2 Department of Molecular Diagnostics, Sun Yat-Sen University Cancer Center, Guangzhou, 510060 China; 3 Department of Pathology, Sun Yat-Sen University Cancer Center, Dongfeng Road, Yuexiu District, No. 651, Guangzhou, 510060 China; 4 Department of Pathology, First Affiliated Hospital of Southern University of Science and Technology, Second Clinical Medical College of Jinan University, Shenzhen People’s Hospital, Shenzhen, 518020 China; 5 Department of Pathology, The First Affiliated Hospital, Sun Yat-sen University, No. 58, Zhongshan Road II, Guangzhou, 510080 China; 6 Department of Pathology, Guangzhou Concord Cancer Center, Guangzhou, 510045 China

**Keywords:** Chondromesenchymal hamartoma, Ribs, Adult, Infant, Pathological diagnosis

## Abstract

**Background:**

Chondromesenchymal hamartoma of the chest wall is a rare, benign disease that usually presents at birth or in early infancy. It typically involves one or more ribs, forming a unilateral or bilateral extrapleural mass. Patients may be asymptomatic or complain of mild respiratory distress depending on tumor size and location. To the best of our knowledge, only two of the approximately 100 cases reported so far are adults.

**Case presentation:**

We present two cases of chondromesenchymal hamartoma. The first case involved the left fifth rib in a 24-year-old male, in close proximity to the fifth vertebral body in the left posterior mediastinum, mimicking a posterior mediastinal tumor on imaging. The tumor was excised via thoracoscopy and the patient had an uneventful postoperative course. The second case was that of a 5-month-old boy, who had a tumor involving the left fifth and sixth ribs which caused thoracic cage collapse. Following *en bloc* resection of the tumor and the involved rib segments, the patient was transferred to the intensive care unit for treatment of pulmonary infection and disseminated intravascular coagulation (DIC). He was discharged from the hospital in stable condition 11 days later. On histopathology, the tumor was found to be a chondromesenchymal hamartoma with immature spindle-shaped mesenchymal cells, plate-like hyaline cartilage, areas of woven bone formation, endochondral ossification and calcification, osteoclastic giant cells, and secondary aneurysmal bone cysts.

**Conclusions:**

Although the presently reported cases have morphological characteristics similar to previously reported ones, they had distinct radiological and clinical characteristics. Patient 1 is only the third report of an adult with chondromesenchymal hamartoma. His case was characterized by its radiological appearance mimicking a posterior mediastinal tumor. Patient 2 represents the first documentation of DIC as a postoperative complication following excision of a chondromesenchymal hamartoma. We present these two cases to provide clinicopathological insights regarding this extremely rare tumor that are relevant to both pathologists and clinicians.

## Background

Chondromesenchymal hamartoma of the chest wall presents at birth or in early infancy as an intraosseous expansile mass involving the ribs. It has an incidence of about 0.03% among primary bone tumors and shows male predominance; approximately 100 cases have been reported worldwide [1]. The tumor is composed of a disorganized admixture of cartilaginous components, spindle cell fascicles, woven bone, and hemorrhagic cysts. Surgical resection is the appropriate treatment and careful follow-up is necessary for early recognition of complications. In some cases, the aggressive appearance of the tumor may prompt unnecessary extended surgery with chest wall reconstruction, which may lead to complications such as trunk deformity and scoliosis [2]. There is no specific immunohistochemical (IHC) marker for this unusual disease. Histologic examination is generally adequate to establish the diagnosis, given the unique morphologic features. Imaging studies offer important clues; however, image-based diagnosis may be difficult, especially with atypical patient age or tumor location. The present case reports illustrate the need for a high suspicion index for chondromesenchymal hamartoma in similar cases.

## Case presentations

### Case 1: Chondromesenchymal hamartoma in a 24-year-old male mimicking a posterior mediastinal tumor

**Fig. 1:**
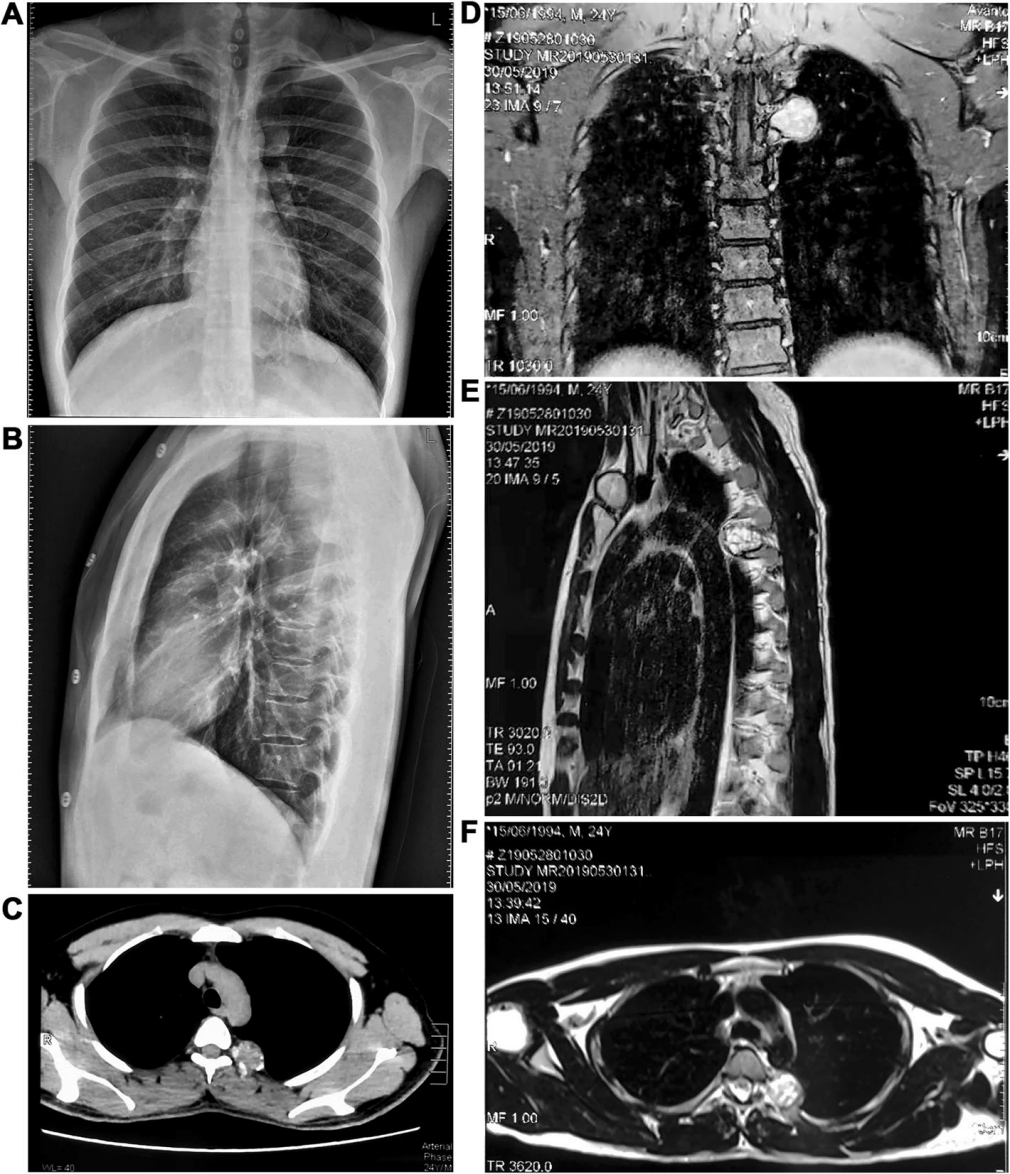
Radiological appearance of chondromesenchymal hamartoma of the chest wall in a 24-year-old adult. **a**, **b** DR suggested a left posterosuperior mediastinal mass and bronchitic changes. **c** CT revealed a benign expansile lesion in the posterior part of the left fifth rib with interior punctate calcifications. **d**-**f** MRI revealed a well-circumscribed expansile heterogeneous soft mass closely adjoining the fifth vertebral body in the left posterior mediastinum. DR, digital radiography; CT, computed tomography; DR, digital radiography; MRI, magnetic resonance imaging

A chondromesenchymal hamartoma of the chest wall was incidentally discovered on the imaging studies of a 24-year-old male who presented with complaints of persistent cough in May 2019. Digital radiography (DR) was suggestive of a left posterosuperior mediastinal mass with bronchial changes (Fig. 1a-b). Computerized tomography (CT) revealed a benign expansile lesion in the posterior part of the left fifth rib with interior punctate calcifications, suggestive of an enchondroma (Fig. 1c). Magnetic resonance imaging (MRI) revealed a well-defined dumbbell shaped lesion with equal T1 and long T2 signals. The lesion measured approximately 32 mm × 25 mm. The expansile heterogeneous soft tissue lesion arising from the left fifth rib closely adjoined the fifth vertebral body in the left posterior mediastinum. The mass was characterized by substantially restricted diffusion and progressive heterogeneous enhancement (Fig. 1d-f). We suspected a chondrogenic or a neurogenic tumor of the left posterior mediastinum. Following preoperative optimization, the mass was thoracoscopically excised; intercostal nerve block and T4**–**6 pedicle internal fixation were performed. The patient had an uneventful recovery and was discharged in stable condition on the third postoperative day.

### Case 2: Chondromesenchymal hamartoma in a 5-month-old boy with postoperative disseminated intravascular coagulation

**Fig. 2:**
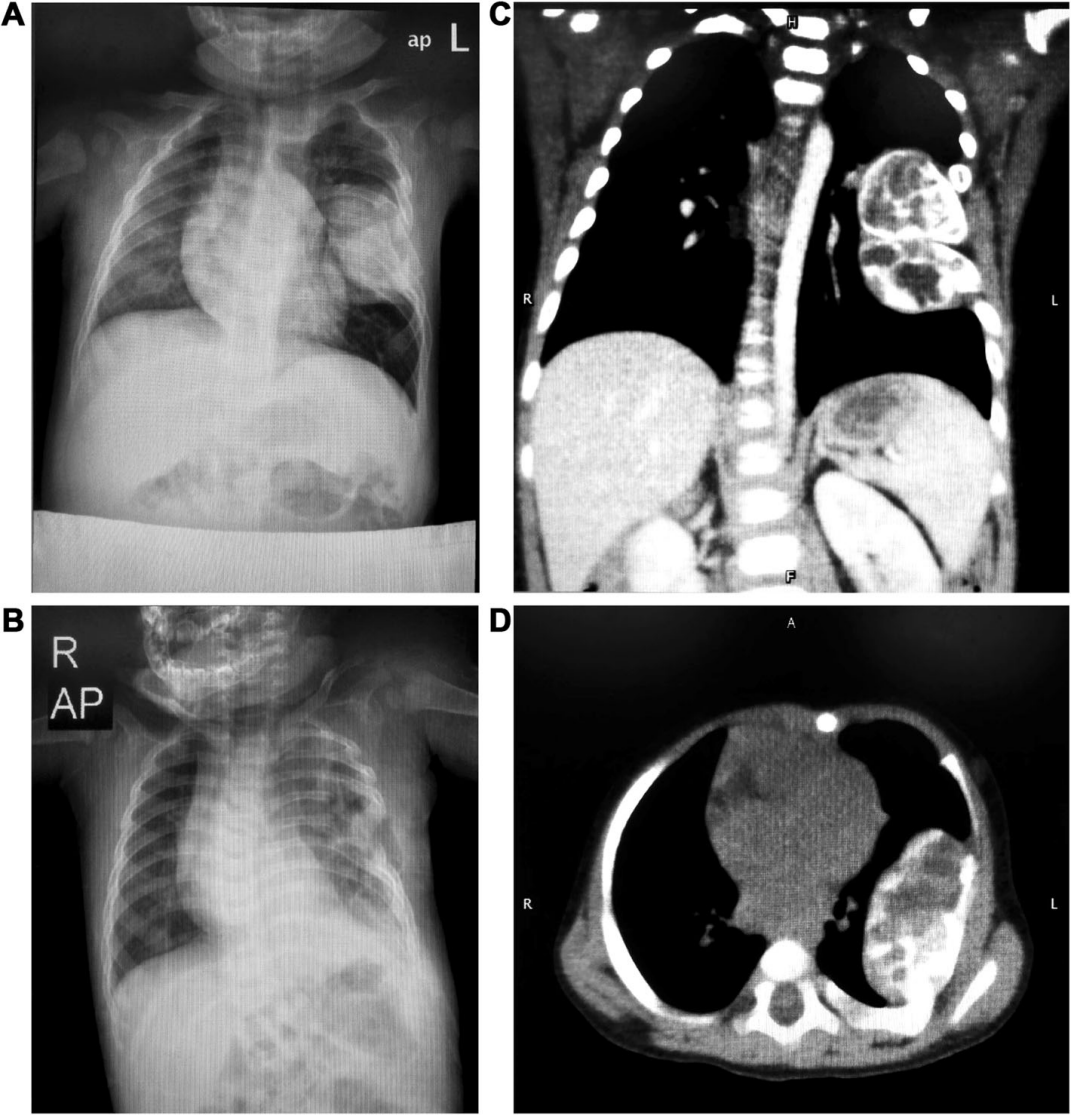
Imaging findings of chondromesenchymal hamartoma of the chest wall in a 5-month-old infant. **a** DR revealed a soft tissue mass in the left middle lung field, accompanied by collapse of the adjacent thoracic cage. **b** DR revealed postoperative loss of parts of the left fifth and sixth ribs. **c**, **d** CT revealed a benign solid-cystic lesion with multiple speckled and cord-like high-density shadows, arising from the axillary segment of the left fifth and sixth ribs; the corresponding cortical and medullary cavities of the ribs were involved. DR, digital radiography; CT, computed tomography

A 5-month-old boy was admitted to the hospital with an asymptomatic, progressively enlarging painless mass in the left infra-axillary area of the lateral chest wall in August 2015. DR revealed a well-circumscribed soft tissue mass in the left middle lung field, measuring approximately 47 mm × 39 mm, accompanied by collapse of the adjacent thoracic cage and deformation of the left fifth and sixth ribs. The lesion was suspected to be a benign chondrogenic tumor (Fig. 2a-b). CT revealed a benign tumor or tumor-like lesion involving the axillary segments of the left fifth and sixth ribs (Fig. 2c). The corresponding cortical and medullary rib cavities were involved and the mass was solid-cystic with several speckled and cord-like high-density internal shadows (Fig. 2d). There was mild enhancement in the solid areas and lack of enhancement in the cystic areas. Localized emphysema in the left lung field was also observed. Based on these radiographic characteristics and the patient’s age, a preoperative diagnosis of mesenchymal hamartoma was made. Two weeks later, the infant underwent *en bloc* resection of the tumor and the involved rib segments. The marrow cavity was sealed using bone wax and a thoracic tube drain was placed.

Postoperatively, the infant developed fever (maximum temperature, 39.7 °C) with marked elevation of C-reactive protein and procalcitonin levels and white blood cell count. He was transferred to the intensive care unit and started on vancomycin and ceftazidime for a presumptive diagnosis of pulmonary infection. Coagulation function tests were suggestive of disseminated intravascular coagulation (DIC): D-dimer, 5.36 mg/L; antithrombin III, 67.4%; fibrinogen degradation products, 11.3 μg/mL; prothrombin time, 19.4 s (11.0–4.0 s); activated partial thromboplastin time, 62.6 s (25.0–35.0 s); and fibrinogen, 5.38 g/L (2.00–4.00 g/L). Heparin sodium was administered as an anticoagulant and fresh frozen plasma was transfused to correct coagulation disorders. Subsequently, the infant’s condition improved, the thoracic tube drain was removed 7 days after surgery, and the infant was discharged from the hospital on the 11th postoperative day.

## Pathological findings

### Case 1

**Fig. 3:**
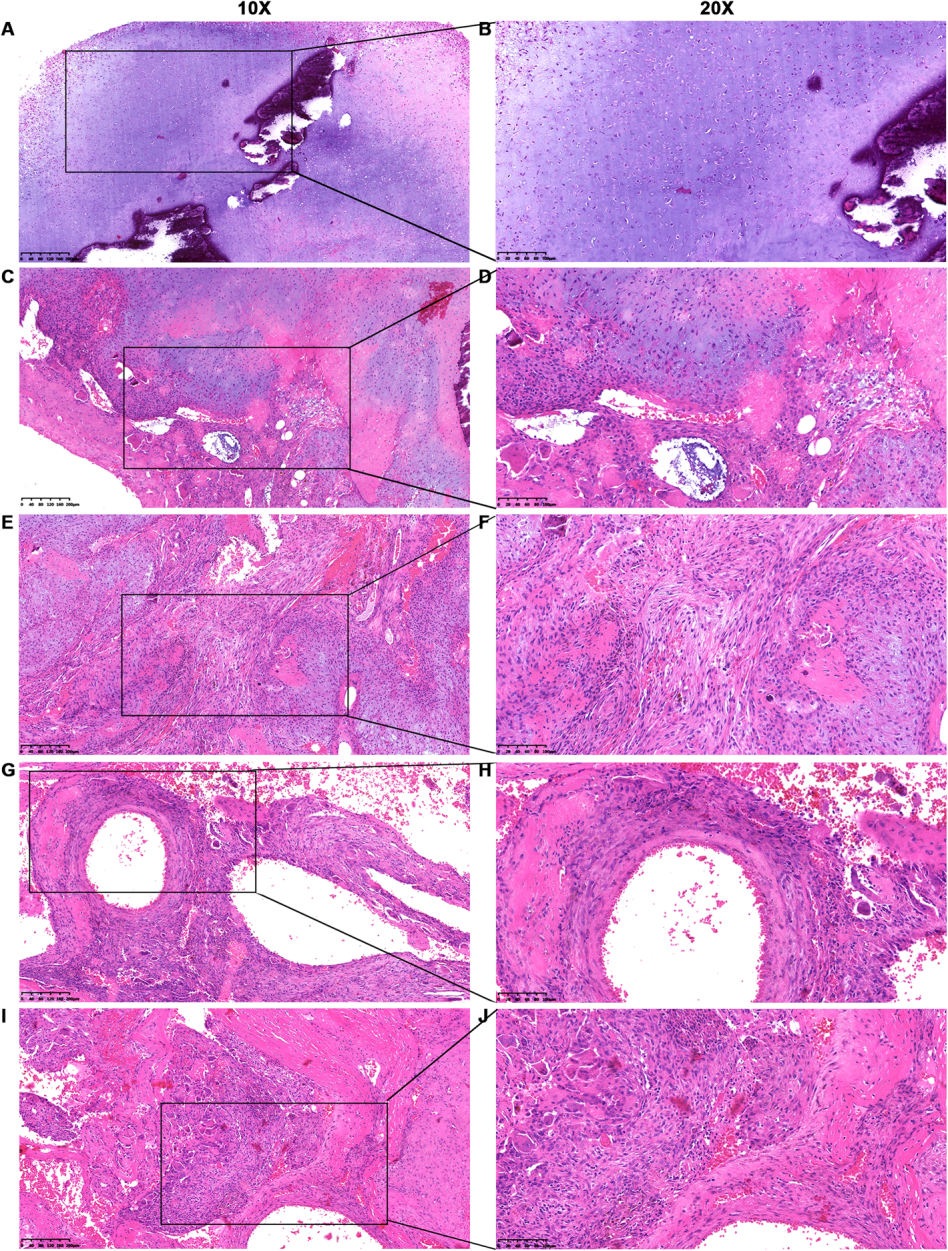
Pathological characteristics of the chondromesenchymal hamartoma (HE staining) in case 1. **a**, **b** Hyaline cartilage with endochondral calcification. **c**, **d** Hyaline cartilage with endochondral ossification and calcification surrounds the ABC-like area. In the lower left region, the fibrous wall is composed of connective tissue stroma with scattered osteoid trabeculae and osteoclast-like giant cells. **e**, **f** A number of mesenchymal spindle-shaped cells interwoven with multilobulated hyaline cartilage. **g**, **h** Typical secondary ABC structures appearing as cystic spaces containing red blood cells. The fibrous walls are composed of connective tissue stroma with scattered osteoid trabeculae and osteoclast-like giant cells. Ossification frequently appears within the fibrous walls and among the cartilage background. **i**, **j** Disorganized mixture of mesenchymal spindle cells, multinucleated osteoclastic giant cells, endochondral ossification, and hemorrhagic spaces with fibrous cystic walls. Left panel, × 10; right panel, × 20. HE, hematoxylin and eosin; ABC, aneurysmal bone cyst

Macroscopically, the tumor was multilocular and measured approximately 3.5 cm × 2.5 cm × 1.5 cm. Microscopically, the solid area consisted of hyaline cartilage with endochondral calcification (Fig. 3a-b) and ossification and fascicles of mesenchymal spindle-shaped cells (Fig. 3c-f). The cystic portion was composed of aneurysmal bone cyst (ABC)-like structures (i.e. hemorrhagic spaces enclosed by fibrous connective cyst walls with scattered osteoid trabeculae and osteoclast-like giant cells). Moreover, ossification could be frequently observed within the fibrous walls and in the cartilage background (Fig. 3g-h). As the name “chondromesenchymal hamartoma” implies, there are typically several different histological components mixed together in a non-malignant pattern: mesenchymal spindle cells, frequent ossification, and secondary changes such as multinucleated osteoclastic giant cells and hemorrhagic spaces with fibrous cystic walls (Fig. 3i-j).

**Fig. 4:**
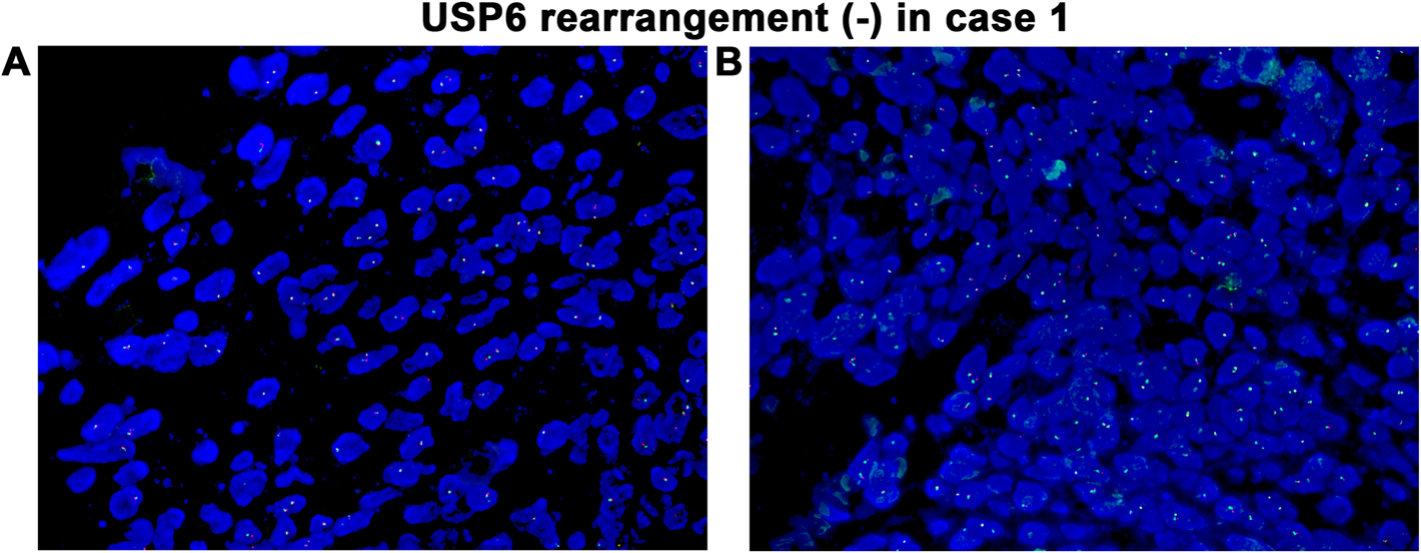
USP6 rearrangement detection by a break-apart probe was negative in case 1. **a**, **b** Two different fields showing how the red and green signals did not break apart but rather appear in the same position or in close positions within the nuclei of the spindle-cell population, which should be interpreted as negative according to the manufacturer’s criteria (× 100)

In addition, fluorescence in situ hybridization (FISH) detection using *USP6* break-apart probes was conducted for case 1. The *USP6* break-apart FISH result was negative (Fig. 4a-b), which strongly rules out primary ABC.

### Case 2

**Fig. 5:**
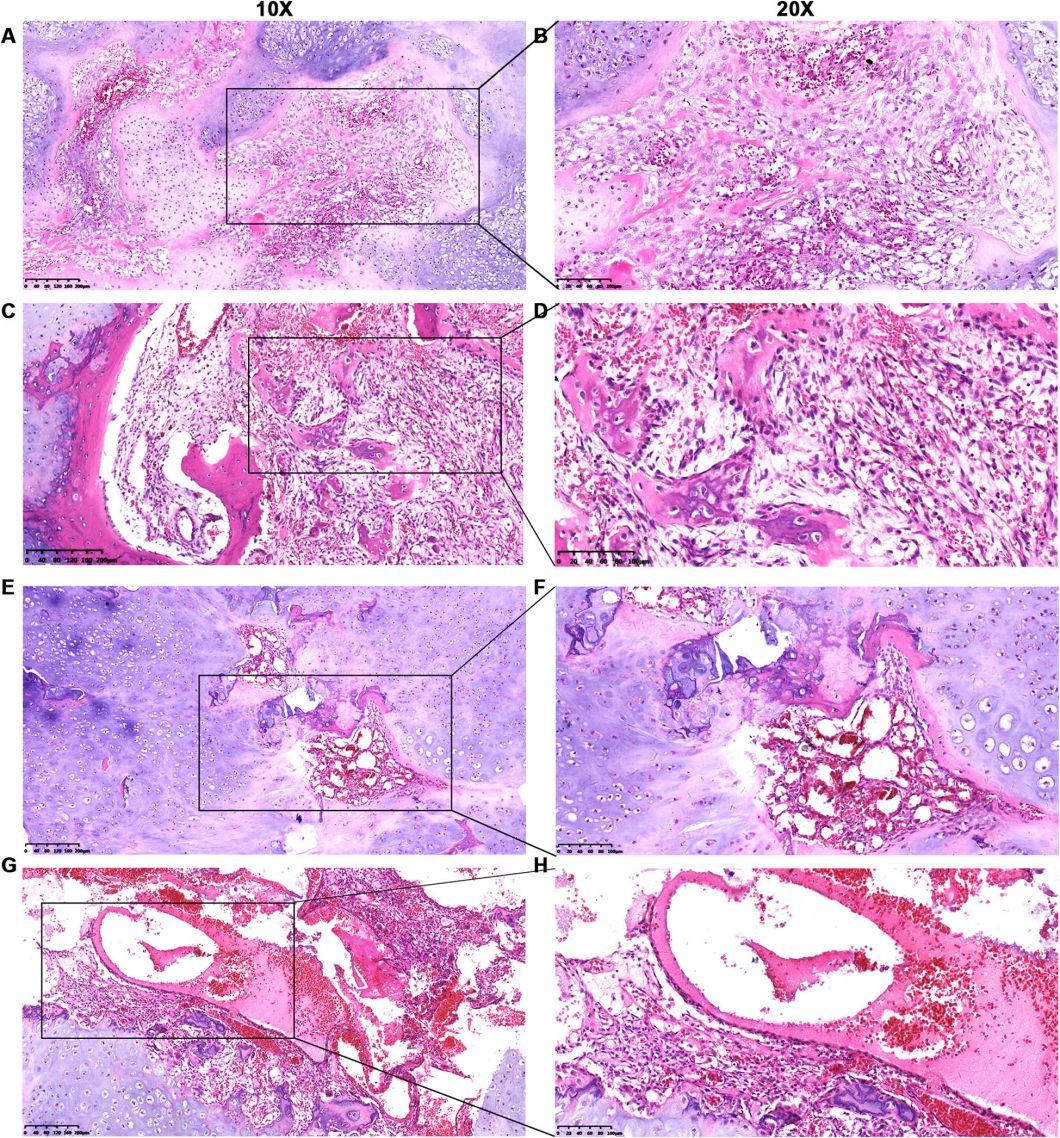
Pathological features of chondromesenchymal hamartoma (HE staining) in case 2. **a**, **b** Fascicles of mesenchymal spindle cells interwoven with multilobulated hypercellular hyaline cartilage. **c**, **d** Disorganized mixture of cartilage, spindle cells, osteoid trabeculae, and osteoclastic giant cells. **e**, **f** Focal myeloid tissues and osteoid trabeculae scattered among hypercellular plate-like cartilage. **g**, **h** Fragmented cartilage mixed with osteoid trabeculae, osteoclast-like giant cells, and blood-filled spaces enclosed by fibrous connective cyst walls. Left panel, × 10; right panel, × 20. HE, hematoxylin and eosin

The tumor measured approximately 5 cm in diameter with focal cystic changes. Microscopically, most of the solid section’s area consisted of fascicles of mesenchymal spindle cells interwoven with multilobulated hypercellular hyaline cartilage (Fig. 5a-b). Scattered woven bones and osteoclastic giant cells were mixed with spindle cells and surrounded by cartilage (Fig. 5c-d). Some myeloid tissues could be observed among the lobulated cartilage (Fig. 5e-f), which confirmed the CT findings of tumor involvement of the corresponding cortical and medullary rib cavities. The cystic area comprised various-sized blood-filled spaces enclosed mostly by fibrous connective tissue, identified as secondary ABCs (Fig. 5g-h). Similarly to case 1, ossification could also be found in the form of spindle-shaped fibroblasts and cartilage in this case.

Collectively, the histological features of both cases were consistent with the diagnosis of chondromesenchymal hamartoma of the chest wall.

### Follow-up information

The 24-year-old male (case 1) had DR examinations at 1 month and 3 months postoperatively, which showed no sign of recurrence, T4–6 pedicle internal fixation could still be observed and the remnant lung had a normal appearance. For case 2, the patient had DR examinations every year since the surgery in 2015, which showed no sign of recurrence and a normal lung appearance. Both patients had neither respiratory issues nor any other newly discovered tumor at the last follow-up on January 30th, 2020.

## Discussion and conclusions

The term “mesenchymal hamartoma” was first proposed in 1979 by McLeod and Dahlin, as it best reflected the benign nature of this lesion composed of disordered but non-neoplastic skeletal tissues [3]. Odell and Benjamin were the first to use the term “mesenchymal hamartoma of the chest wall” in 1986 [4]. Its incidence is estimated to be 1 in 3000 among primary bone tumors or less than 1 per million in the general population [5]. Approximately 100 cases have been described to date, most occurring prenatally or within the first 6 months of life [6]. To the best of our knowledge, only two cases of chondromesenchymal hamartoma have been reported in adults. Bilateral multifocal lesions (CT revealed three masses on the right side and two masses on the left side) were discovered in a 47-year-old man who did not undergo treatment until 13 years later when he developed chest pain [7]. Another asymptomatic left chest wall tumor was discovered incidentally during a complete medical checkup in a 39-year-old woman. The tumor was excised *en bloc* with segments of the 7th and 8th ribs [8]. The age of onset in our case (24 years old) is also quite uncommon, making this only the third adult patient with chondromesenchymal hamartoma reported worldwide.

Chondromesenchymal hamartomas are usually unilateral and are commonly seen on the right side, with a male-to-female ratio of 1.6:1 [9]. A few cases of bilateral lesions have also been reported [1, 7, 10]. Typically, these lesions arise from one or several ribs and their size may range from a few to a dozen centimeters. In most cases, these lesions occur in isolation; however, they are occasionally multifocal [7, 11, 12]. Patients may present with respiratory distress or be asymptomatic. Less common manifestations include scoliosis, chest wall deformity, cough, and fever [1]. One infant died 14 days after birth of severe sepsis secondary to *Pseudomonas aeruginosa* infection and pulmonary insufficiency [13]. Patient 2 in this report is the first documented instance of DIC as a postoperative complication following excision of chondromesenchymal hamartoma. Although this complication may not be directly related to this condition or its surgical treatment, it remains a possibility; therefore, we believe that assessing coagulation function preoperatively is important.

Imaging studies are helpful in determining the site of origin, tumor density, enlargement, and effect on adjacent structures; however, imaging is not considered diagnostic and may be misleading if the tumor location or patient age is atypical [14, 15]. In patient 1, the imaging finding of a paravertebral mass in an adult appeared to mimic a posterior mediastinal tumor and the radiologist suggested the possibility of a neurogenic tumor. Malignant lesions such as congenital neuroblastoma, Ewing’s sarcoma, malignant teratoma, osteosarcoma, or chondrosarcoma cannot be excluded in the presence of cortical erosion, rib destruction, or deformation of adjacent ribs as seen on imaging [16, 17]. Biopsy of the lesion can be complicated by severe bleeding because of disruption of the vascular spaces; therefore, needle biopsy should be performed cautiously [1, 2].

Microscopically, chondromesenchymal hamartomas have immature spindle-shaped mesenchymal cells, plate-like hyaline cartilage, woven bone formation, endochondral ossification and calcification, osteoclastic giant cells, and secondary ABC changes; abnormal mitoses and atypia are not present [18]. Woven trabeculae containing hematopoietic marrow are common, as observed in patient 2. Areas resembling ABC, with osteoclast-like giant cells, blood-filled spaces, hemosiderin-laden macrophages, and fibromembranous septa, are specific for chondromesenchymal hamartoma. It has been proposed that the formation of an ABC is secondary to intraosseous arteriovenous fistula formation [19]. IHC staining may demonstrate the presence of S-100 protein in cartilaginous areas [4]. To the best of our knowledge, no current molecular genetic tests are available to assist in the diagnosis of chondromesenchymal hamartoma [10].

The differential diagnosis of chondromesenchymal hamartoma includes tumoral and non-tumoral lesions involving the ribs that are common in infants and children, including primary ABC, chondrosarcoma, enchondroma, osteochondroma, fibrous dysplasia, and osteofibrous dysplasia (OFD) [14]. Primary ABC and chondromesenchymal hamartoma both show cystic areas; however, they lack solid cartilage nodules and component diversity. It is noteworthy that ABC could be secondary to various bone tumors, including giant cell tumors, chondroblastomas, fibrous histiocytomas, chondromyxoid fibromas, fibrous dysplasia, and osteosarcoma [20]. Chondrosarcoma is primarily a tumor of adulthood and older age [21]. It is characterized by high cellularity, presence of host bone entrapment, and absence of host bone encasement. Chondrosarcoma is characterized by mild-to-moderate atypical chondrocytes, varying in size and shape, and containing enlarged, hyperchromatic nuclei. Myxoid changes or chondroid matrix liquefaction is a common feature of chondrosarcomas [22]. Enchondroma is a benign hyaline cartilage neoplasm arising within the medullary bone cavity; normal bone marrow elements may also be observed between its nodules, as seen in patient 2. Noticeably, enchondroma often appears as pale blue on hematoxylin and eosin staining owing to its high matrix proteoglycan content and it is less diverse in terms of histological components than chondromesenchymal hamartoma. Osteochondroma originates from the bone surface and possesses a distinctive three-layer structure of perichondrium, cartilage, and bone. The outer layer is a fibrous perichondrium that is continuous with the periosteum, below which is a hyaline cartilage cap with endochondral ossification. Similar to chondromesenchymal hamartoma, fibrous dysplasia can occur in the ribs, contain a cartilaginous component with endochondral ossification, and have secondary changes including ABC-like areas and multinucleated osteoclastic giant cells. However, fibrous dysplasia is mainly composed of bland fibroblastic cells and irregular trabeculae of woven bone; mesenchymal cells and plate-like hyaline cartilage are not its main components [22]. OFD mostly involves cortical bone of the anterior mid-shaft of the tibia during infancy and childhood; it is composed of fragments of woven bone rimmed by lamellar bone layers laid down by well-defined osteoblasts [22]. Although secondary ABC and multinucleated giant cells may be seen in OFD, there is an absence of cartilage. Lung hamartoma, which is the most common cartilage-containing benign lung tumor, should also be taken into consideration in the differential diagnosis. Since this tumor is composed of tissues that are normally present in the lung, the presence of normal bronchial epithelium could be a valuable clue [23]. Unlike chondromesenchymal hamartoma, lung hamartoma is mostly found in the lung parenchyma or within the bronchus and may only secondarily involve the ribs [24].

Molecular diagnostic techniques have recently emerged as independent diagnostic tools to improve diagnostic accuracy and reduce interobserver variability; many characteristic genetic alterations have been identified in bone tumors [25]. *USP6* and/or *CDH11* rearrangements are found in 69% of primary ABCs, but not in secondary ABC [26]. *IDH1* (R132C; R132H) or *IDH2* (R172S) mutations may occur in enchondromas, atypical cartilaginous tumor/grade 1 central chondrosarcomas, grade 2/3 central chondrosarcomas, and dedifferentiated chondrosarcomas; however, they are absent in osteochondromas [27, 28]. *MDM2* and *CDK4* are amplified in low-grade central osteosarcomas and periosteal osteosarcomas, as demonstrated by FISH/IHC [29]. K36M mutations in *H3F3B* appear in about 95% of chondroblastomas, while G34W/L mutations in *H3F3A* are found in 92% of giant cell tumors of the bone [30]. It is likely that molecular markers will increasingly play an important role in improving the diagnosis and treatment of bone tumors.

The treatment strategy for chondromesenchymal hamartoma involves one of two main approaches: conservative management for asymptomatic patients and surgical treatment for patients with respiratory distress caused by mass compression. A case of spontaneous regression of a chest wall hamartoma in an infant was reported [31]. In most cases, surgical resection is chosen irrespective of symptoms. Secondary surgery may be required following incomplete resections [9]. Patients with significant upper airway obstruction may need permanent tracheotomy [32]. A third management option, radiofrequency thermoablation (RFT), a relatively noninvasive technique performed under CT guidance, was performed in a 6-month-old girl [33]. RFT causes coagulative necrosis in the lesion, which is gradually reabsorbed. This method avoids damage to the adjacent normal bone and is well tolerated in children, thereby decreasing the risk of severe postoperative complications [34].

Herein, we reported two extremely rare cases of chondromesenchymal hamartoma. Although the lesions in these cases were morphologically similar to previously reported cases, they had distinct radiological and clinical characteristics. To the best of our knowledge, case 1 is only the third report of an adult patient with chondromesenchymal hamartoma. This patient was suspected of having a posterior mediastinal tumor on radiology. Case 2 is the first documentation of DIC as a postoperative complication of chondromesenchymal hamartoma. This report may raise awareness regarding the presentation, diagnosis, and management of chondromesenchymal hamartoma among pathologists, radiologists, and clinicians.

## Data Availability

The datasets used and/or analyzed during the current study are available from the corresponding author on reasonable request.

## Abbreviations

ABC: Aneurysmal bone cyst
CT: Computerized tomography
DIC: Disseminated intravascular coagulation
DR: Digital radiography
FISH: Fluorescence in situ hybridization
IHC: Immunohistochemical
MRI: Magnetic resonance imaging
OFD: Osteofibrous dysplasia
RFT: Radiofrequency thermoablation
